# Efficacy of Bilateral Erector Spinae Block in Patients Undergoing Posterior Spine Fusion Surgeries: A Comparative Randomised Controlled Trial

**DOI:** 10.7759/cureus.55366

**Published:** 2024-03-01

**Authors:** Suresh Kumar, Arish BT, Eashwar Neelakandan, Ranjan RV, Sivakumar Segaran, Prince Solomon

**Affiliations:** 1 Anaesthesiology, Pondicherry Institute of Medical Sciences, Pondicherry, IND; 2 Anaesthesiology, Sri Manakula Vinayagar Medical College and Hospital, Pondicherry, IND; 3 Orthopaedics, Pondicherry Institute of Medical Sciences, Pondicherry, IND

**Keywords:** opioid consumption, hemodynamic stability, pain scores, perioperative analgesia, posterior spine surgeries, erector spinae block

## Abstract

Introduction

During spine surgeries, various levels of tissue injury can result in varying hemodynamic responses and significant postoperative pain. Perioperative pain management is essential to controlling hemodynamic responses and postoperative pain management. Erector spinae plane (ESP) blocks can help alleviate this pain by blocking the dorsal rami of the spinal nerve. This study aims to evaluate the efficacy of ESP by assessing the perioperative opioid requirement, hemodynamic parameters, and visual analogue score (VAS) during the postoperative period.

Methods

In this study, 56 patients underwent elective posterior spine fusion surgeries under conventional anaesthesia and were allocated into two groups: 28 patients were included in the conventional group (Group C) and 28 patients in the ESP group (Group E). Group C patients received 20 ml of 0.9% sodium chloride (NaCl) on each side, and Group E patients received 20 ml of 0.25% bupivacaine + 4 mg dexamethasone on each side under ultrasound sonography guidance. Postoperative pain was assessed using the VAS score. The hemodynamic parameters during the intraoperative period, the time for the first opioid analgesia requirement until 24 hours in the postoperative period, and the amount of cumulative opioid consumption during the perioperative period were observed.

Results

Postoperative VAS was lower in Group E (P < 0.001). There were significant differences in hemodynamic parameters: heart rate (P < 0.045), systolic blood pressure (P < 0.002), diastolic blood pressure (P < 0.003), and mean arterial pressure (P < 0.002) at the time of incision in Group E. Intraoperative opioid requirements at the time of incision (P < 0.036), 60th minutes (P < 0.023), 120th minutes (P < 0.023), and postoperative opioid requirements at the first hour (P < 0.001), sixth hour (P < 0.004), 14th hour (P < 0.025), 20th hour (P < 0.009), and 24th hour (P < 0.025) had lower opioid requirements in Group E than Group C.

Conclusion

ESP block is a more site-specific dorsal rami block with a better perioperative hemodynamic profile, a part of multimodal analgesia intraoperatively, and excellent postoperative analgesia with fewer postoperative opioid requirements in multilevel spine fusion surgeries.

## Introduction

Globally, there is an increased incidence of spine pathologies with varying clinical manifestations, which include back pain, motor weakness, radiating pain to the legs, and entrapment neuropathies. Chronic pain, disability, diminished motor function, and poor quality of life are the probable outcomes in these untreated patients [1].

The management of these pathologies typically involves conservative management and/or operative procedures. Conservative strategies like physiotherapy, steroid injections, opioids, gabapentinoids, and non-steroidal analgesics provide short-term pain relief. Surgical procedures like discectomy, laminectomy, spine fusion, and decompression surgeries are more effective in moderate-to-severe diseases that do not respond to conservative management. During spine surgeries, various levels of tissue injuries, which include skin, subcutaneous tissue, muscle, periosteum, and bone, can result in varying hemodynamic responses and significant postoperative pain. Poor perioperative pain management can lead to increased opioid consumption, delayed mobilisation, and pulmonary and thromboembolic complications, thereby increasing morbidity and mortality [2].

In the majority of patients, severe pain was reported within four hours of surgery, and it could last up to postoperative day 3 [2]. Opioids are an important class of drugs used in the pharmacological approach. Other systemic analgesics include non-steroidal anti-inflammatory drugs (NSAIDs), ketamine, gabapentin, pregabalin, and dexmedetomidine, which are usually part of a multimodal analgesic regimen [2]. Interventional procedures encompass local infiltration techniques, neuraxial blocks, paravertebral blocks, retrolaminar blocks, and erector spinae plane (ESP) blocks.

In 2016, Forero et al. introduced the ESP block, a novel interfascial plane block that demonstrated remarkable efficacy in chronic neuropathic pain patients. This block provided analgesia by blocking the dorsal rami nerves with minimal complications [3]. Other practitioners also found the ESP block to be a highly effective and viable alternative to paravertebral and epidural blocks for acute postoperative pain in thoracotomy in terms of safety, simplicity, and broad dermatomal coverage [4].

To our knowledge, limited studies have explored the efficacy of ESP block in spine surgeries involving various levels. Therefore, we conducted this study to assess the effectiveness of ESP blocks in posterior spine fusion surgeries at different levels.

The primary objective is to assess postoperative pain by using a visual analogue score (VAS) for 24 hours after spine surgeries, comparing the effect of the ESP block between a general anaesthesia group and a conventional general anaesthesia group. The secondary objectives include comparing the hemodynamic parameters between the two groups, determining the first opioid requirement within 24 hours postoperatively, and evaluating the cumulative opioid consumption during intraoperative and postoperative periods (up to 24 hours).

This article was previously presented by a final-year postgraduate student at the 69th annual national conference of the Indian Society of Anaesthesiologists, dated November 22-27, 2022.

## Materials and methods

This single-centre, double-blinded randomised controlled clinical trial was conducted after obtaining institutional ethics committee clearance from Pondicherry Institute of Medical Sciences, Puducherry, India (approval number: RC/2020/94) between 2021 and 2022. The trial was registered in the Clinical Trials Registry-India (CTRI) (CTRI 2021/07/034708) before patient enrolment. We enrolled 56 patients who underwent posterior spine fusion surgery in our institution. Patients with American Society of Anaesthesiologists (ASA) physical status I and II, aged 18-65 years, of both sexes were included. We excluded patients who refused to participate, as well as those with BMI > 35 kg/m, coagulopathy disorders, allergies to the study drug, and undergoing chronic treatment with opioids.

Considering the mean difference in the VAS (control group 3.37 ± 1.35 and study group 2.40 ± 0.89) from the previous study by Yayik et al. [2], with a 95% CI and 80% power, the sample size calculated was 25 in each group. After block randomization and considering 10% attrition of participants, a sample size of 28 in each group was assigned (total n = 56).

All participants were allocated randomly into two groups, conventional (Group C) and ESP (Group E), by using computer-generated block randomization with a block size of seven and eight patients in each block. Group C patients received conventional general anaesthesia with a normal saline (0.9% NaCl) block, and Group E received conventional general anaesthesia with a bilateral ultrasound-guided erector spinae block.

All patients were kept on fasting overnight and premedicated with oral alprazolam 0.25 mg, pantoprazole 40 mg, and metoclopramide 10 mg on the day of surgery. On arrival at the OR, ASA monitoring standards such as ECG, non-invasive blood pressure, pulse oximeter, and baseline parameters were recorded. After securing intravenous access, all patients were preoxygenated for three minutes. General anaesthesia was induced with midazolam 30 mcg/kg IV, fentanyl 2 mcg/kg IV, and propofol 2 mg/kg IV, and muscle relaxation was achieved with vecuronium bromide 0.1 mg/kg IV. After securing the airway with a flexometallic tube of appropriate size, patients were positioned prone to all safety measures. Anaesthesia was maintained with oxygen, nitrous oxide (50:50), and sevoflurane, and muscle relaxation was maintained with intermittent doses of vecuronium bromide (0.02 mg/kg). Under strict aseptic precautions, a high-frequency linear or curvilinear ultrasound probe was placed longitudinally over the spine. The probe slid from the medial to lateral directions, approximately 3 cm from the midline, to identify the erector spinae muscles and the transverse process. Using a 23-gauge Quincke spinal needle, an in-plane approach was used, and the needle tip was placed between the erector spinae muscles and the corresponding transverse process.

For cervical-level surgeries, the needle was introduced at the C7-T1 level and directed in the caudal to the cranial manner. For thoracic and lumbar levels, the needle was introduced one segment above the centre of the proposed instrumentation and directed cranially to the caudal. After confirming the correct needle tip position by hydro dissection, Group C patients received 40 ml of normal saline (0.9% NaCl) (20 ml on each side), and Group E patients received a total 40 ml mixture of 0.25 % bupivacaine + 8 mg dexamethasone (20 ml on each side) below the erector spinae muscles on both sides, respectively. A paracetamol infusion of 15 mg/kg was administered before skin incision and postoperatively every six hours for 24 hours. Heart rate, blood pressure, and mean arterial pressure (MAP) were monitored every 15 minutes until the end of the surgery. Fentanyl 25 mcg IV bolus was administered and documented for any increase in the hemodynamic parameters of >20% of baseline intraoperatively as a rescue analgesic. After surgery, neuromuscular blockade was reversed with neostigmine 50 mcg/kg + glycopyrrolate 10 mcg/kg IV, and the patient was extubated. The VAS score was recorded for every hour during the first 12 hours and the second hour thereafter for 24 hours, and the first opioid analgesic dose of tramadol 50 mg slow IV was given when VAS ≥ 4, and the total postoperative opioid consumption was also monitored for the first 24 hours for both groups. Intraoperative heart rate, blood pressure, and MAP were recorded every 15 minutes after the intervention, and additional intraoperative opioid consumption was documented. All data points were taken from the trend tables on the monitor.

The student unpaired t-test was used to analyse age, weight, height, BMI, and hemodynamic parameters. Sex, ASA, site of surgery, number of levels and different levels of distribution, and intraoperative and postoperative opioid consumption were analysed using a chi-square test. The VAS score was expressed using the Mann-Whitney U test and the repeat analysis of variance test. A P-value of < 0.05 was considered statistically significant.

## Results

**Figure 1:**
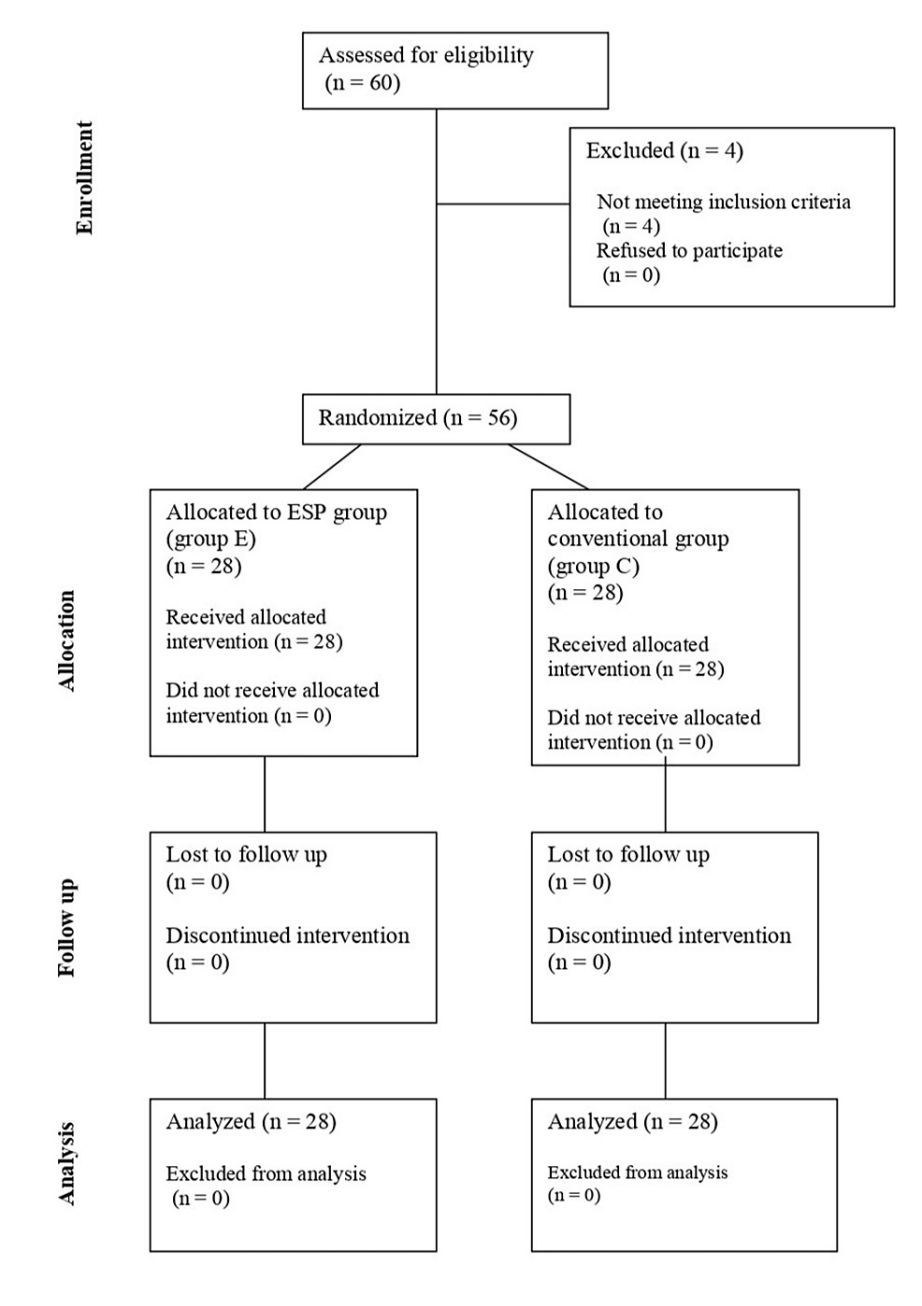
Consort flowchart ESP: erector spinae plane

Fifty-six patients with ASA status I and II who had spine surgeries were randomly allocated into two groups, Group C and Group E, of 28 patients each (Figure 1).

As seen in Table 1, the mean age was found to be 50.29 ± 11.55 in Group C and 48.29 ± 11.84 in Group E. There were 12 female patients and 16 male patients in both groups. The average anthropometry measures in Group C were 64.32 ± 9.71 kg, 160.39 ± 7.06 cm, 25.04 ± 3.88 kg/m^2^, and in Group E were 65.36 ± 10.81 kg, 164.11 ± 10.40cm, 24.04 ± 3.67 kg/m^2^. There were nine ASA I patients and 19 ASA II patients in both groups, respectively. All the demographic data were comparable, and there was no significant difference between the two groups.

**Table 1:** Demographic and clinical characteristics of the participants BMI: body mass index; ASA: American Society of Anesthesiologists; Group C: conventional group; Group E: erector spinae plane group

S. No.	Variables			Group C	Group E	P-value
1.	Age (years)			50.29 ± 11.55	48.29 ± 11.84	0.525 (student unpaired t-test)
2.	Gender		Female	12	12	0.001 (chi-square test)
			Male	16	16	
3.	Weight			64.32 ± 9.71 kg	65.36 ± 10.81 kg	0.708 (student unpaired t-test)
4.	Height			160.39 ± 7.06 cm	164.11 ± 10.40 cm	0.125 (student unpaired t-test)
5.	BMI			25.04 ± 3.88 kg/m^2^	24.04 ± 3.67 kg/m^2^	0.474 (student unpaired t-test)
6.	ASA	I		9	9	1.0 (chi-square test)
		II		19	19	

**Figure 2:**
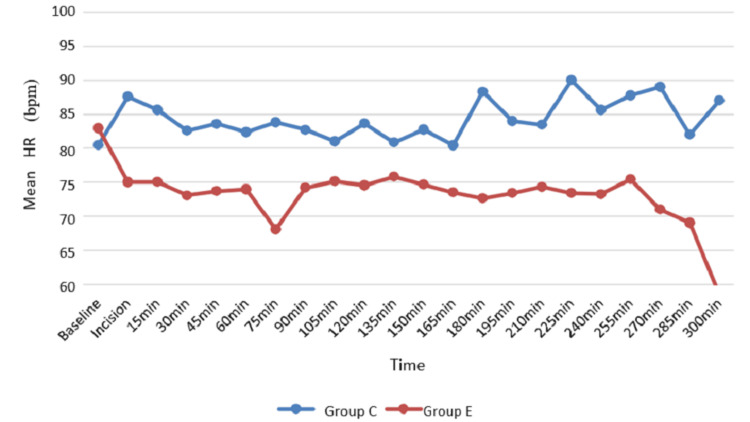
Mean HR among groups HR: heart rate; bpm: beats per minute; min: minute; Group C: conventional group; Group E: erector spinae plane group

The mean heart rate was 80.43 ± 12.32 to 90.00 ± 10.02 beats per minute (bpm) in Group C and 58.50 ± 9.19 to 82.93 ± 11.10 bpm in Group E. There was a statistically significant difference between the two groups at the time of incision, 15th to 90th minute, 120th, 180th, 225th, and 240th minute (Table 2, Figure 2).

**Table 2:** Mean HR among groups HR: heart rate; bpm: beats per minute; min: minute; Group C: conventional group; Group E: erector spinae plane group

Time	Group	N	Mean HR (bpm)	Standard deviation	P-value	Mean difference	95% confidence interval of the difference	
							Lower	Upper
Baseline	Group C	28	80.46	11.783	0.424	-2.464	-8.599	3.67
	Group E	28	82.93	11.102				
Incision	Group C	28	87.57	16.256	0.007	12.571	3.538	21.605
	Group E	28	75	17.438				
15 min	Group C	28	85.64	14.205	0.004	10.607	3.456	17.758
	Group E	28	75.04	12.414				
30 min	Group C	28	82.57	12.77	0.004	9.536	3.181	15.89
	Group E	28	73.04	10.858				
45 min	Group C	28	83.57	12.261	0.003	9.893	3.588	16.197
	Group E	28	73.68	11.245				
60 min	Group C	28	82.32	15.497	0.021	8.393	1.322	15.464
	Group E	28	73.93	10.281				
75 min	Group C	28	83.79	14.543	0.003	15.75	5.766	25.734
	Group E	28	68.04	21.867				
90 min	Group C	28	82.71	14.102	0.019	8.514	1.455	15.574
	Group E	25	74.2	11.464				
105 min	Group C	25	80.96	13.612	0.152	5.81	-2.233	13.853
	Group E	20	75.15	13.011				
120 min	Group C	24	83.63	10.623	0.013	9.125	2.043	16.207
	Group E	18	74.5	11.592				
135 min	Group C	19	80.84	12.773	0.239	5.019	-3.494	13.531
	Group E	17	75.82	12.335				
150 min	Group C	28	82.74	12.238	0.069	8.09	-0.663	16.843
	Group E	28	74.65	13.43				
165 min	Group C	14	80.43	12.321	0.148	6.929	-2.612	16.469
	Group E	14	73.5	12.24				
180 min	Group C	13	88.31	10.226	0.001	15.665	7.122	24.208
	Group E	14	72.64	11.325				
195 min	Group C	8	84	9.725	0.069	10.6	-0.922	22.122
	Group E	10	73.4	13.302				
210 min	Group C	7	83.43	7.345	0.089	9.129	-1.566	19.823
	Group E	10	74.3	13.158				
225 min	Group C	5	90	10.025	0.02	16.6	3.236	29.964
	Group E	10	73.4	12.616				
240 min	Group C	5	85.6	5.367	0.026	12.378	1.774	22.982
	Group E	9	73.22	12.637				
255 min	Group C	4	87.75	6.238	0.142	12.35	-5.637	30.337
	Group E	5	75.4	14.605				
270 min	Group C	2	89	1.414	0.098	18	-6.033	42.033
	Group E	4	71	15.253				
285 min	Group C	2	82	2.828	0.121	13	-5.724	31.724
	Group E	4	69	12.193				
300 min	Group C	2	87	2.828	0.119	28.5	-31.476	88.476
	Group E	2	58.5	9.192				

The mean systolic blood pressure (SBP) ranges from 141.75 mmHg to 116.00 in Group C and 123.61 to 96.75 mmHg in Group E. There was a statistically significant difference between the two groups at the time of incision: 15th to 75th minute, 120th, 210th, 225th, and 285th minute (Table 3).

**Table 3:** Mean SBP among groups SBP: systolic blood pressure; mmHg: millimetre of mercury; SD: standard deviation; min: minute; Group C: conventional group; Group E: erector spinae plane group

Time	Group	N	Mean SBP (mmHg)	SD	P-value	Mean difference	95% confidence interval of the difference	
									Lower	Upper
							Baseline	Group C	28	118.75	13.517	0.211	-4.857	-12.558	2.844
Group E	28	123.61	15.171												
				Incision	Group C	28		132.61	24.696	0	25.429					14.617	36.24
Group E	28	107.18	13.944														
					15 min	Group C	28	119.93	17.073			0.006	11.714	3.468	19.961		
Group E	28	108.21	13.456												
				30 min		Group C	28	120.43	20.973	0.002	15.857					6.171	25.543
Group E	28	104.57	14.49														
					45 min	Group C	28	121.93	18.154			0.019	12.214	2.127	22.302		
Group E	28	109.71	19.471												
				60 min		Group C	28	123.79	27.263	0.028	13.214					1.539	24.89
Group E	28	110.57	13.825														
					75 min	Group C	28	122.89	19.458			0.002	16.124	6.472	25.776		
Group E	26	106.77	15.794												
				90 min		Group C	28	116.04	18.8	0.157	6.836					-2.729	16.401
Group E	25	109.2	15.869														
					105 min	Group C	25	117.84	16.193			0.084	8.49	-1.204	18.184		
Group E	20	109.35	15.849												
				120 min		Group C	23	126.74	23.148	0.001	19.906					8.297	31.515
Group E	18	106.83	13.049														
					135 min	Group C	19	114.32	15.955			0.26	5.551	-4.292	15.394		
Group E	17	108.76	13.07												
				150 min		Group C	19	124	29.494	0.076	14.059					-1.601	29.718
Group E	17	109.94	14.541														
					165 min	Group C	14	115.79	12.789			0.286	5.786	-5.13	16.702		
Group E	14	110	15.171												
				180 min		Group C	13	126.23	23.41	0.081	14.731					-1.958	31.42
Group E	14	111.5	17.844														
					195 min	Group C	8	125.13	17.117			0.055	17.825	-0.397	36.047		
Group E	10	107.3	19.259												
				210 min		Group C	7	124.57	12.726	0.041	17.371					0.842	33.901
Group E	10	107.2	19.234														
					225 min	Group C	5	133.8	21.73			0.034	29.8	3.311	56.289		
Group E	9	104	9.552												
240 min	Group C	5	123.2	9.654	0.022	17.95	3.083	32.817
	Group E	8	105.25	14.665				
									255 min	Group C	4	141.75	13.326	0.004	47.5	26.942	68.058
Group E	4	94.25	3.304														
				270 min	Group C	2	128.5	26.163		0.332	31.75	-187.368	250.868				
Group E	4	96.75	4.856										
					285 min	Group C	2	115.5	4.95					0.045	16.5	0.714	32.286
Group E	4	99	6.831														
				300 min		Group C	2	116	5.657	0.13	17	-23.487	57.487				
Group E	2	99	1.414										

The mean diastolic blood pressure (DBP) ranges from 83.75 to 71.43 mmHg in Group C and 77.61 to 59.25 mmHg in Group E. There was a statistically significant difference between the two groups at the time of incision: 15th, 30th, 75th, 120th, 225th, and 255th minutes (Table 4).

**Table 4:** Mean DBP among groups DBP: diastolic blood pressure; min: minute; mmHg: millimetre of mercury; SD: standard deviation; Group C: conventional group; Group E: erector spinae plane group

Time	Group	N	Mean DBP (mmHg)	SD	P-value	Mean difference	95% confidence interval of the difference	
									Lower	Upper
							Baseline	Group C	28	74.07	9.165	0.147	-3.536	-8.356	1.285
Group E	28	77.61	8.825												
				Incision	Group C	28		78	13.469	0.013	8.214					1.837	14.592
Group E	28	69.79	10.042														
					15 min	Group C	28	78.07	11.382			0.015	7.286	1.47	13.101		
Group E	28	70.79	10.293												
				30 min		Group C	28	75.61	12.072	0.01	7.571					1.853	13.29
Group E	28	68.04	9.012														
					45 min	Group C	28	77.07	11.956			0.077	5.929	-0.668	12.525		
Group E	28	71.14	12.654												
				60 min		Group C	28	76.89	14.5	0.1	5.321					-1.061	11.703
Group E	28	71.57	8.382														
					75 min	Group C	28	77.04	11.998			0.019	7.151	1.203	13.099		
Group E	26	69.88	9.726												
				90 min		Group C	28	71.43	12.677	0.866	-0.531					-6.833	5.77
Group E	25	71.96	10.134														
					105 min	Group C	25	74.24	9.001			0.422	2.34	-3.494	8.174		
Group E	20	71.9	10.073												
				120 min		Group C	23	77.83	10.933	0.005	8.833					2.854	14.812
Group E	18	69	8.239														
					135 min	Group C	19	71.68	7.024			0.392	2.214	-2.978	7.406		
Group E	17	69.47	8.14												
				150 min		Group C	19	75.95	11.616	0.18	5.183					-2.521	12.886
Group E	17	70.76	11.11														
					165 min	Group C	14	71.64	7.662			0.923	0.357	-7.182	7.896		
Group E	14	71.29	11.276												
				180 min		Group C	13	79.54	12.19	0.129	7.396					-2.306	17.097
Group E	14	72.14	12.265														
					195 min	Group C	8	77.13	12.147			0.417	4.925	-7.627	17.477		
Group E	10	72.2	12.813												
				210 min		Group C	7	80.43	7.185	0.164	7.529					-3.449	18.506
Group E	10	72.9	13.739														
					225 min	Group C	5	82.4	9.529			0.027	14.178	2.034	26.321		
Group E	9	68.22	9.471												
240 min	Group C	5	74.2	4.207	0.214	4.7	-3.156	12.556
	Group E	8	69.5	8.536				
									255 min	Group C	4	83.75	8.958	0.005	24.5	10.765	38.235
Group E	4	59.25	6.185														
				270 min	Group C	2	82.5	17.678		0.351	20.25	-134.141	174.641				
Group E	4	62.25	2.062										
					285 min	Group C	2	72	2.828					0.06	8.75	-0.784	18.284
Group E	4	63.25	3.202														
				300 min		Group C	2	72	2.828	0.069	11	-2.837	24.837				
Group E	2	61	1.414										

**Figure 3:**
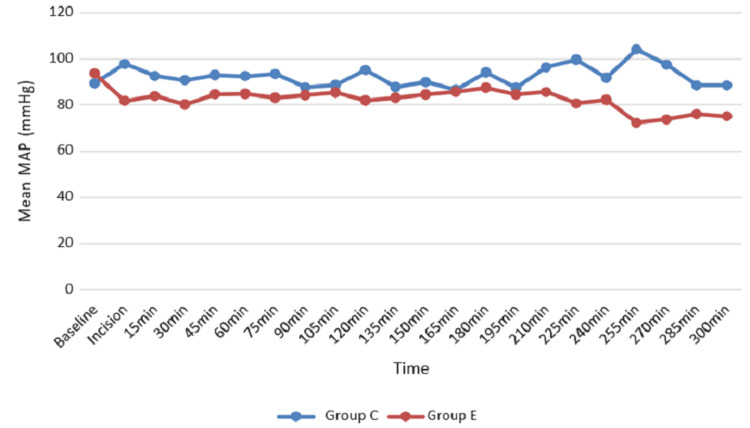
MAP among groups MAP: mean arterial pressure; mmHg: millimetres of mercury; min: minute; Group C: conventional group; Group E: erector spinae plane group

The average MAP in Group C was 86.50 to 104.00 mmHg, and in Group E it was 72.25 to 93.64 mmHg. There was a significant difference in MAP between two groups at the time of incision: 15th to 45th minute, 75th, 120th, 225th, 255th, and 300th minute (Figure 3).

The mean additional intraoperative fentanyl consumption was 1.22 mg in Group C and 125 mcg in Group E (Table 5). The difference between the two groups was statistically significant at the time of incision (0.036), 60th minute (0.023), and 120th minute (0.023).

**Table 5:** Intraoperative cumulative additional opioid dose among groups In Group C, we repeated fentanyl 42 times, and the cumulative dose was 1.05 mg. In Group E, we repeated fentanyl only five times, and the cumulative dose was 125 mcg Group C: conventional group; Group E: erector spinae plane group; mg: milligrammes; mcg: micrograms

Intraoperative cumulative fentanyl dose	Group C	Group E
Total number of repeated doses	42	5
Cumulative dose	1.05 mg	125 mcg

**Figure 4:**
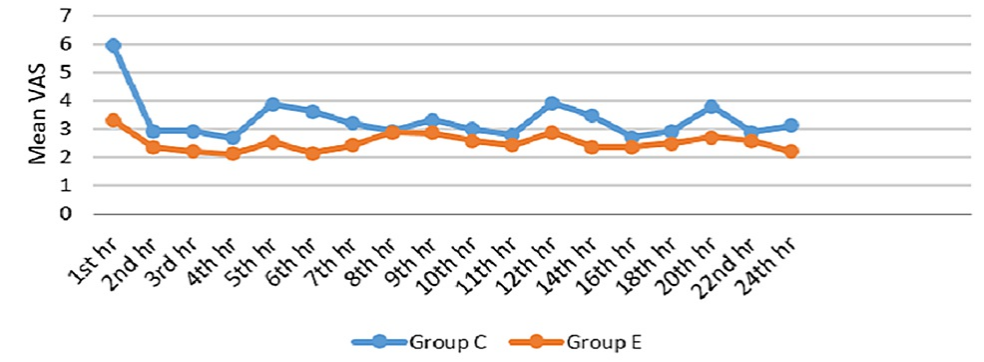
Mean VAS among groups VAS: visual analogue scale; hr: hour; Group C: conventional group; Group E: erector spinae plane group

The average VAS score was 5.96 to 2.68 in Group C and 3.32 to 2.11 in Group E. There was a statistically significant difference in VAS between the two groups from the first to the seventh hour, 12th, 14th, 16th, 20th, and 24th hour postoperatively (Table 6, Figure 4).

**Table 6:** Mean VAS among groups VAS: visual analogue score; hr: hour; VAS ranges: 0-10; Group C: conventional group; Group E: erector spinae plane group; IQR: interquartile range

Time	Group	N	Mean	Standard deviation	Median	IQR	P-value	Mean difference	95% confidence interval of the difference	
									Lower	Upper
1^st^ hr	Group C	28	5.96	2.236	6.500	3.0	<0.001	2.643	1.534	3.752
	Group E	28	3.32	1.887	3.000	1.0				
2^nd^ hr	Group C	28	2.93	0.979	3.000	1.0	0.001	0.571	0.086	1.057
	Group E	28	2.36	0.826	2.000	1.0				
3^rd^ hr	Group C	28	2.93	0.979	3.000	1.0	<0.001	0.714	0.295	1.134
	Group E	28	2.21	0.499	2.000	1.0				
4^th^ hr	Group C	28	2.68	1.056	2.000	1.0	0.010	0.571	0.125	1.018
	Group E	28	2.11	0.497	2.000	0.0				
5^th^ hr	Group C	28	3.89	2.347	3.000	4.0	0.007	1.357	0.273	2.441
	Group E	28	2.54	1.621	2.000	1.0				
6^th^ hr	Group C	28	3.61	1.571	3.000	2.0	<0.001	1.464	0.827	2.101
	Group E	28	2.14	0.525	2.000	0.0				
7^th^ hr	Group C	28	3.21	1.343	3.000	1.0	0.006	0.786	0.235	1.336
	Group E	28	2.43	0.504	2.000	1.0				
8^th^ hr	Group C	28	2.93	1.016	3.000	1.0	0.471	0.036	-0.568	0.640
	Group E	28	2.89	1.227	3.000	1.0				
9^th^ hr	Group C	28	3.32	1.744	3.000	1.0	0.251	0.464	-0.329	1.258
	Group E	28	2.86	1.145	2.500	1.0				
10^th^ hr	Group C	28	3.00	1.515	3.000	1.0	0.222	0.429	-0.239	1.096
	Group E	28	2.57	.879	2.000	1.0				
11^th^ hr	Group C	28	2.79	1.134	2.500	1.0	0.116	0.357	-0.187	0.901
	Group E	28	2.43	0.879	2.000	1.0				
12^th^ hr	Group C	28	3.93	1.844	3.000	4.0	0.024	1.036	0.181	1.890
	Group E	28	2.89	1.286	2.000	1.0				
14^th^ hr	Group C	28	3.46	1.710	3.000	3.0	0.005	1.107	0.415	1.799
	Group E	28	2.36	0.559	2.000	1.0				
16^th^ hr	Group C	28	2.71	0.937	3.000	1.0	0.017	0.357	-0.159	0.874
	Group E	28	2.36	0.989	2.000	0.0				
18^th^ hr	Group C	28	2.93	1.152	3.000	1.0	0.073	.429	-0.122	0.979
	Group E	28	2.50	0.882	2.000	1.0				
20^th^ hr	Group C	28	3.79	1.729	3.000	4.0	0.013	1.071	0.282	1.861
	Group E	28	2.71	1.150	2.000	1.0				
22^nd^ hr	Group C	28	2.89	0.956	3.000	1.0	0.105	0.321	-0.171	0.814
	Group E	28	2.57	0.879	2.000	1.0				
24^th^ hr	Group C	28	3.11	1.315	3.000	2.0	0.001	0.893	0.353	1.433
	Group E	28	2.21	0.499	2.000	0.0				

The cumulative postoperative opioid requirement was 5700 mg in Group C and 2000 mg in Group E (Table 7). There was a significant difference in postoperative opioid consumption between the two groups at the first, fifth, sixth, 14th, 20th, and 24th hours.

**Table 7:** Mean postoperative opioid requirement among groups In Group C, we repeated tramadol 114 times, and the cumulative dose was 5700 mg. In Group E, we repeated tramadol only 40 times, and the cumulative dose was 2000 mg Group C: conventional group; Group E: erector spinae plane group; mg: milligrammes; mcg: micrograms

Postoperative cumulative tramadol dose	Group C	Group E
Total number of repeated doses	114	40
cumulative dose	5700 mg	2000 mg

The distribution of postoperative first opioid requirement was found to be higher in Group C than in Group E at the first, fifth, sixth, 14th, 20th, and 24th hour (Table 8). There was no significant difference in the additional opioid or NSAID consumption between the two groups at any given time.

**Table 8:** Distribution of the postoperative first opioid requirement Group C: conventional group; Group E: erector spinae plane group; hr: hour

Time	Group	N	Postoperative opioid required	No postoperative opioid required	P-value
						1^st^ hr	Group C	28	22	6	<0.001
							Group E	28	4	24	
2^nd^ hr	Group C	28	2	26	0.491						
	Group E	28	0	28	
						3^rd^ hr	Group C	28	2	26	0.491
Group E	28	0	28								
				4^th^ hr	Group C		28	2	26	0.491	
Group E	28	0	28							
					5^th^ hr	Group C	28	9	19		0.019
Group E	28	2	26								
				6^th^ hr		Group C	28	8	20	0.004	
Group E	28	0	28							
					7^th^ hr	Group C	28	4	24		0.111
Group E	28	0	28								
				8^th^ hr		Group C	28	3	25	1	
Group E	28	4	24							
					9^th^ hr	Group C	28	5	23		1
Group E	28	5	23								
				10^th^ hr		Group C	28	3	25	1	
Group E	28	3	25							
					11^th^ hr	Group C	28	3	25		1
Group E	28	2	26								
				12^th^ hr		Group C	28	11	17	0.146	
Group E	28	6	22							
					14^th^ hr	Group C	28	8	20		0.025
Group E	28	1	27								
				16^th^ hr						Group C		28	2	26	1
Group E	28	1	27												
						18^th^ hr	Group C	28	5							23	0.705
Group E	28	3	25														
					20^th^ hr					Group C	28	13	15	0.009			
Group E	28	4	24														
				22^th^ hr			Group C	28	4	24	1						
Group E	28	4	24											
					24^th^ hr	Group C	28	8	20	0.025	
Group E	28	1	27								

## Discussion

Spine surgeries result in intraoperative hemodynamic disturbances due to sympathetic stimulation and severe postoperative pain due to large incisions and tissue handling, which cannot be tolerated by all patients. Adequate perioperative analgesia in spine surgery plays a vital role in attenuating surgical stress response, better recovery, and early ambulation in the postoperative period.

The routine practice is to use a pharmacological approach rather than interventional methods. Drugs such as opioids, NSAIDs, and ketamine, as well as non-opioids like gabapentin, pregabalin, dexmedetomidine, and others, can be used as part of multimodal analgesia. Increasing the dose of these analgesics can reduce the pain, but they are associated with other side effects like delayed recovery, postoperative desaturation, postoperative nausea, vomiting, urinary retention, etc. On the other hand, we have interventional methods like local infiltration, neuraxial techniques, paravertebral blocks, and ESP blocks that will effectively block the afferent pain signals, as they not only reduce the opioid and inhalational anaesthetic requirements but also postoperative complications.

In literature, the majority of ESP blocks were performed solely on the lumbar level [5-7]. We assessed the effectiveness of ESP block at various levels of posterior spine surgeries (cervical, thoracic, and lumbar) by parameters such as intraoperative opioid consumption, hemodynamics, time duration for the first rescue analgesia postoperatively, and the total amount of opioid requirement for the first 24 hours postoperatively.

The needle orientation is a key factor in the ESP block because it determines the drug spread. Kanna et al. assessed the drug spread in caudal to cranial needle orientation at the level of the T1 vertebra for posterior cervical spine surgeries, which resulted in excellent cephalad spread of the drug and superior perioperative pain relief [8]. Similarly, in our study for the cervical level, the needle was directed from the caudal to the cephalad from one segment below the skin incision. We found that there was a good local anaesthetic (LA) spread of cephalad, which resulted in good pain relief.

Finnerty et al. performed ESP block at the T5 level for minimally invasive thoracic surgeries, and they directed the needle in a cranial to caudal manner. They found that the ESP block was superior to the serratus anterior plane block and no longer needed for rescue analgesia in the ESP group [9]. Likewise, in our study, at the thoracic level, the needle was introduced in the cranial to caudal direction from one vertebra above the level of the surgery, resulting in a diffuse spread of LA and a good analgesic effect.

Yayik et al. performed ESP block at the L3 level for lumbar spine surgeries, in which they introduced the needle in a cranial to caudal direction, resulting in adequate spread through the entire lumbar level [2]. In our study, the needle was oriented in the cranial to the caudal direction at the L3-L4 level, resulting in excellent spread of the drug and a greater analgesic effect.

Concerning age, gender, height, weight, BMI, and ASA grading, there were no statistically significant differences, and the two groups were comparable.

Singh et al. assessed the postoperative pain score with a numerical rating scale in patients who received bilateral ESP blocks for lumbar spine surgeries. They found that the pain score was significantly higher in the control group immediately after surgery and at six hours than in the ESP block group, and the satisfaction score was higher in the ESP block group (P < 0.0001) [10].

Yayik et al. found that the VAS scores were significantly lower in the ESP group than in the control group in patients who underwent lumbar spinal decompression surgery [2].

Similarly, in our study, the visual analogue score was assessed between two groups at various intervals. The Group E patients had lower VAS scores compared to Group C for the first seven hours and at 12, 14, 16, 20, and 24 hours postoperatively, and it was statistically significant (P < 0.001). This signifies that there was no requirement for analgesics postoperatively until seven hours in the ESP block group when compared to the conventional group. The site-specific blockade of the dorsal rami of the spinal nerves helps to achieve greater analgesia for patients in the postoperative period.

Ghamry et al. showed that there was a significant increase in HR and MAP in the general anaesthesia group rather than general anaesthesia with ESP block at various time intervals in patients who underwent posterior lumbar interbody fusion [11].

Based on an observational study by Zhang et al. on volunteers, the ESP block produced cutaneous sensory loss from T6-T9 and was probably due to the involvement of the dorsal rami of spinal nerves [12].

Likewise, in our study, heart rate was significantly low, especially at the time of incision: 15th to 90th, 120th, 180th, 225th, and 240th minutes in the ESP block (P = 0.045). The SBP was significantly lower at the time of incision: 15th to 75th, 120th, 225th, and 285th minutes. Similarly, DBP was significantly low in Group E at the time of incision: 15th, 30th, 75th, 120th, 225th, and 255th minutes. MAP was found to be higher in Group C patients than Group E patients, and the difference between the two groups was statistically significant at the time of incision: 15th to 45th, 75th, 120th, 225th, 255th, and 300th minutes. The controlled hemodynamics were probably due to superior analgesia provided by effectively blocking the dorsal rami of spinal nerves.

Goel et al., in their study, found that ESP group patients had low fentanyl consumption compared to the conventional general anaesthesia group [13].

Correspondingly, in our study, Group C patients had higher opioid requirements than Group E patients at the time of incision: 60th and 120th minutes. Higher-quality analgesia provided by the ESP block reduced opioid consumption intraoperatively. With the ESP block, the amount of opioid consumption and the associated side effects were found to be reduced.

Similarly, Wahdan et al. explained in their study that morphine consumption was higher in the control group than in the ESP block group in the postoperative period [14].

The rescue analgesia in our study was tramadol 50 mg intravenously. Both groups received 1 gm of acetaminophen every eighth hour until 24 hours as part of multimodal analgesia. We found that tramadol consumption was significantly higher in Group C than in Group E at different periods. The longer duration of postoperative analgesia could be due to the effect of the dexamethasone additive in the block [15].

The main limitation of our study was that we could not evaluate the extent of sensory blockade as we performed the procedure after GA. Patient-controlled analgesia was not used, which would have helped us to refine the time intervals and quantify the exact amount of opioid requirements. In our study, we did not standardise the number of patients at a particular spine level. Recovery questionnaires were not assessed to determine the long-term quality of life after the ESP block.

## Conclusions

The ESP block is a site-specific dorsal ramus block with a better perioperative hemodynamic profile. When used as a part of multimodal analgesia, it provides excellent intraoperative and postoperative analgesia with fewer postoperative opioid requirements in multilevel spine fusion surgeries.
